# IGF expression in HPV-related and HPV-unrelated human cancer cells

**DOI:** 10.3892/or.2014.3329

**Published:** 2014-07-11

**Authors:** JULIA DURZYŃSKA, ELISABETH BARTON

**Affiliations:** 1 Department of Molecular Virology, Faculty of Biology, Institute of Experimental Biology, Adam Mickiewicz University, 61-614 Poznań, Poland; 2 Department of Anatomy and Cell Biology, School of Dental Medicine, University of Pennsylvania, Philadelphia, PA 19104, USA

**Keywords:** cancer cells, IGF splice variants, human papillomavirus, nuclear and cytoplasmic fractions, western blotting, quantitative RT-PCR

## Abstract

The human *Igf-1* gene not only produces insulin-like growth factor-I (IGF-I), but also different carboxy-terminal extensions, known as E peptides, through alternative splicing. We and others have shown that human Eb peptide (hEb) derived from *Igf-1* has intrinsic biological activity and is localized to nuclei of transfected cells. Since hEb actions can complement the activity of IGF-I itself, the aim of the present study was to compare IGF-I isoforms at the endogenous protein and transcript level in cancer cell lines, including HeLa, U2OS, HepG2 and K562 cells. Quantitative real-time PCR (qRT-PCR) using *Igf-1* isoform specific primers was performed to determine expression patterns, using *β-actin* as a reference gene. The overall relative *Igf-1* transcript level was different across the cell lines, with ~80-fold higher expression in K562 (130.2±31.2) than in U2OS cells (1.7±1.1). The relative copy number of *Igf-1b* was the highest in HepG2 (69.9±28.6) and K562 cells (28.3±6.7), whereas the relative copy numbers of *Igf-1a* and *Igf-1c* were significantly higher in K562 cells compared to all other cell lines. Immunoblotting using total cell lysates, cytoplasmic and nuclear fractions were carried out to determine the level and distribution of IGF-I proteins. K562 cells exhibited the highest level of hEb in total cell lysates and nuclear fractions and no cell lines displayed hEb in the cytoplasmic fractions. In contrast, IGF-IA was the highest in HeLa cells and was enriched only in the cytoplasmic fraction. Since relatively low IGF-1A transcript level but relatively high pro-IGF-1A protein level is plausible, we hypothesized that these transcripts could be processed with higher efficiency and/or the protein product may be stabilized by viral HPV oncogenes in HeLa cells. We assert that while it is important to analyze *Igf-1* transcript level, it may be more relevant to determine the IGF isoforms at the protein level.

## Introduction

Alternative splicing (AS) of pre-mRNA allows many gene products with different functions to be produced from a single coding sequence. This is a mechanism by which higher-order diversity is generated in compact human genomes (>9×10^4^ different proteins vs. <25×10^3^ genes) (1–2). However, errors in pre-mRNA splicing leading to altered gene expression patterns can result in cell transformation and cancer. Affected proteins can include transcription factors, cell signal transducers and components of the extracellular matrix (2). One of these transducers is insulin-like growth factor-I (IGF-I), a key player in the somatotropic axis (3). It is a 70 amino acid long peptide regulating processes such as cell growth, proliferation, differentiation in almost all cell types (4). It has been considered for many years that for acquisition of its biological activity IGF-I needs to be fully processed at the transcript and protein level in order to become mature and active hormone, although in the last two decades, activities to pro-IGF and resulting E-peptides were assigned (5–8). The *Igf-1* gene structure is very complex and the number of alternative splicing products is impressive; in humans, six exons can be spliced to two IGF classes (I and II depending on which promoter is used) and three isoforms are present in each class, A, B and C depending on exons 4, 5 and 6 combination fused to exon 3 and 4 coding for mature peptide (9). The combination of the last three *Igf-1* exons is called C-terminal extension or E-peptide (10,11). These E-peptides are either cleaved by proteases to release mature IGF or stay attached and together with ‘mature IGF sequence’ to form pro-IGF-I (A, B or C). It has been recently demonstrated that pro-IGF-1A form is as potent as mature IGF-1 to activate IGF-1R and is a predominant form present in muscle (12). Another level of complexity in the IGF-1 activity is glycosylation of IGF-1A isoform. A gly-pro-IGF-1A can be generated since only C-terminal extension of an A form can be glycosylated in rodents and humans. This particular aspect has not yet been studied extensively.

The longest pro-IGF-1 isoform is human pro-IGF-1B composed of 147 amino acids as a product of *Igf-1* gene splicing pattern exon 1/2-exon 3-exon 4-exon 5 (13). It can be cleaved to mature IGF-I and E-peptide of 70 and 77 amino acids, respectively. It is of note that in case of IGF-1B isoform, the C-terminal extension is even bigger than the mature IGF product. There have been a very limited number of studies concerning human Eb-peptide, which may be due, in part, to the lack of an appropriate and specific antibody. Previous studies used only hybrid proteins and immunodetection of human Eb peptide was based on either anti-GFP or anti-RFP antibodies (13,14), which is a less precise approach as compared to the one specifically targeting an antigen of interest. Afforded detection of endogenous IGF-I is always better than relying on transfection models and overexpression.

The aim of the present study was to analyze human IGF-I isoforms at the protein and transcript level, taking advantage of oligonucleotides specific for each form, as well as newly generated antibodies for the A isoform (15) and B isoform produced specifically for this study. We compared IGF-I levels in 4 cancer cell lines: HepG2, K562, HeLa and U2OS. There are multiple advantages of these cell lines from our study perspective. First, they are all immortalized human cells that can grow and divide indefinitely under optimal culture conditions. Second, they exhibit different levels of IGF-I production. HepG2 and K562 cells are known to have high IGF-1 expression level; the former originated from liver being the main source of IGF-1 in the circulation and the latter have one of the highest levels of total IGF-1 among all cell lines (www.proteinatlas.org) (16,17). Both cell lines were expected to show detectable levels of endogenous IGF-1B at the protein level in western blotting experiments. Third, U2OS cells produce low levels of IGF-1 and can be considered as a cell line very poor in IGF-1 (‘IGF negative cell line’), whereas the HeLa line is of considerable interest since it is transformed with human papillomavirus 18 (HPV-18). Fourth, influence of viral oncogenes on splicing patterns and crucial cellular proteins has already been described and we sought to verify if such interference would be possible for IGF-1.

## Materials and methods

### Cell culture, cell lines and cell fractionation

The following cell lines were used: *homo sapiens* cervix adenocarcinoma transformed with human papillomavirus HPV18 (HeLa), *homo sapiens* osteosarcoma (U2OS), hepatocellular carcinoma (HepG2) and human immortalized myelogenous leukemia (K562). In addition, U2OS stable monoclonal cell lines were used expressing RFP-hEb fusion protein as previously described (14). All cell lines with the exception of K562 were cultured in DMEM containing 10% fetal bovine serum (FBS) supplemented with 100 U/ml ampicillin and 100 U/ml streptomycin. Stable cell lines were supplemented with G-418 at 200 μg/ml instead of ampicillin/streptomycin, whereas K562 cells, known to produce high level of overall IGF-1 (www.proteinatlas.org) (16,17), were cultured in a special medium (RPMI, 10% FBS, penicillin and streptomycin at 100 U/ml, non-essential amino acid, 200 mM L-glutamine, 10 mM HEPES and 1 mM sodium puryvate). Before harvesting, cells were washed with PBS and trypsinized using standard procedures (K562 cells do not require trypsinization). All culture reagents were purchased from Life Technologies (Grand Island, NY, USA).

Protease and phosphatase inhibitor cocktails were added to all buffers according to the manufacturer’s suggestions (P8340 and P5726, Sigma Chemical Co., St. Louis, MO, USA). Briefly, cells were transferred from culture dishes to Eppendorf tubes and an estimated packed cell volume (PCV) of 200 μl was mixed with 5× PCV of hypotonic lysis buffer (10 mM Hepes pH=7.9, 1.5 mM MgCl_2_ and 10 mM KCl) supplemented with 0.5 mM DTT and protease inhibitors. Cells were incubated on ice for 15 min allowing them to swell. They were centrifuged for 5 min at 420 × g and resuspended in 400 μl of isotonic lysis buffer (10 mM Tris-HCl, pH 7.5, 2 mM MgCl_2_, 3 mM CaCl_2_ and 0.3 M sucrose with the same supplements described above). Cells were transferred into a glass tissue grind tube and ground on ice slowly with 15–20 up and down strokes using a type B pestle. Trypan blue solution was added to a small portion of cells and cell lysis was confirmed under light microscope. Disrupted cells were centrifuged for 20 min at 10,000 × g. The supernatant was transferred to a fresh tube as a cytoplasmic fraction. For protocol details, http://www.sigmaaldrich.com/technical-documents/protocols/biology/nuclear-protein-extraction.html. The crude nuclei pellet and total cell extracts were resuspended in RIPA buffer [20 mM Tris-HCl (pH 7.5), 150 mM NaCl, 1 mM Na_2_EDTA, 1 mM EGTA, 1% NP-40, 1% sodium deoxycholate, 2.5 mM sodium pyrophosphate, 1 mM β-glycerophosphate, 1 mM Na_3_VO_4_ and 1 μg/ml leupeptin) containing also 0.5 mM DTT and other protease inhibitors (P8340). The protein contents of all preparations were measured using the Bradford procedure (Bio-Rad protein assay; Bio-Rad Laboratories, Hercules, CA, USA).

### RNA extraction, cDNA synthesis and qRT-PCR

Total RNA was isolated from cells using TRIzol reagent (Invitrogen, Life Technologies, Grand Island, NY, USA). RNA concentration was evaluated by reading optical density in 260 nm (SpectraMax M5 plate reader; Molecular Devices, Sunnyvale, CA, USA).

Equal amounts (1 μg) of total RNA from each sample were subjected to single-strand reverse transcription using GenAmp RNA PCR kit with both random hexamers and oligo d(T)16 (Applied Biosystems, Foster City, CA, USA). The resultant cDNA was utilized for quantitative RT-PCR (qRT-PCR) using the Applied Biosystems 7300 real-time PCR System and reagents (Power SYBR Green PCR Master Mix). Oligonucleotides listed in Table I specific to *Igf-1* isoforms were first checked for accuracy by PCR (PuReTaq Ready-To-Go PCR Beads; GE Healthcare, Buckinghamshire, UK) and resolved in 1.5% agarose gel stained with ethidium bromide. Before qRT-PCR was run on test samples, serial dilutions of cDNA obtained from K562 cells were prepared (10-fold dilutions over 7 logs) and the amplification efficiency for all pairs of oligonucleotides designed according to sequence no. NT_019546.15 was calculated from the slope(s) of the standard curve according to the following formula: E = 10(−1/slope) −1 (Table I). For an efficiency of 100%, the slope is −3.32. A good reaction should have an efficiency between 90 and 110%, which corresponds to a slope between −3.58 and −3.10. R^2^ values were ≥0.99 and only 1 pair of nucleotides showed a lower value of 0.98, which was still acceptable. All oligonucleotides used in this study met these gold standards for qRT-PCR parameters. Details are shown in Table I. Regrading qRT-PCR, all samples were loaded in duplicate in 96-well plates. Expression of *β-actin* was used to control for cDNA content. Fold change (relative copy number) was calculated by comparing ΔCt values for each *Igf-1* splice variant, total *Igf-1* and *β-actin*.

### E-peptide synthesis, immunization and antibody purification

A 45 amino acid sequence of human Eb peptide was synthesized to generate an Eb specific antibody. The sequence encompassed the last portion of C-terminus of human IGF-1B isoform (GWPKTHPGGEQKEGTEASLQIRGKKKEQRRE IGSRNAECRGKKGK) and it is not present in any other IGF isoform. Human Eb (based from GenBank: X56774.1) was synthesized using a Thuramed TETRAS 106 peptide synthesizer at a 10 mg scale and purified via HPLC >95% (Shimadzu LC-10AT pump). The final product was confirmed by MALDI mass spectrometry (Waters ZQ 2000 mass spectrometer and Shimadzu LCMS-2010EV). Furthermore, the peptide was coupled with keyhole limpet haemocyanin (KLH) through an additional cysteine. The hapten carrier was used in order to elicit a better immune response in immunized rabbits as KLH is a very strong immunogen. A very similar approach was used for production of anti-MGF antibody (18,19). Two New Zealand white male rabbits (NZW), aged 3 months, ~2.5 kg mass, were immunized by four injections of 1 mg of synthetic Eb-peptide-KLH conjugate in 0.5 ml phosphate-buffered saline (PBS), administered subcutaneously at week 0, 4, 8 and 12. The first immunization was performed with the addition of 0.5 ml of complete Freund’s adjuvant. Further immunizations to boost the rabbits were performed with incomplete Freund’s adjuvant (1, 2 and 3 months after first immunization). Blood samples from rabbits were collected every week immediately before (pre-immune) and 8 times after first immunization. Serum was immediately separated from blood. The animals were sacrificed on day 101 and blood was collected by heart puncture. Serum from the last blood collection was used for antibody purification using magnetic beads containing protein A (Novazym, Poznań, Poland) and antibody was eluted and concentration was measured, which was 7.41 mg/ml and 1.52 mg/ml for animal 1 and 2, respectively. The antibody concentration was standardized to 1 mg/ml before further use. The immunization process was carried out according to the guidelines for the use of laboratory animals and the Polish Ethics Committee for animal research.

### Immunoblotting

Equal amounts of protein were subjected to SDS-PAGE using 16.5% Tris/Tricine (Bio-Rad, USA) and transferred to polyvinylidene fluoride membranes (Immobilon-P; Millipore Corporation, Billerica, MA, USA). Membranes were blocked in Tris-buffered saline (TBS) plus 0.1% Tween-20 (TTBS) and 5% non-fat dry milk. Membranes were incubated in primary antibody diluted in 5% milk-TTBS overnight at 4°C. The following primary antibodies were used in the present study: rabbit polyclonal anti-hEb peptide described above (dilution, 1:30,000), rabbit polyclonal anti-IGF-1Ea (dilution, 1:30,000) (15), mouse monoclonal anti-lamin A/C (dilution, 1:1,000) (Cell Signaling Technology, Danvers, MA, USA), rabbit polyclonal anti-DsRed (dilution, 1:1,000) (Clontech Laboratories, Mountain View, CA, USA) and mouse monoclonal anti-α-tubulin (1:4,000) (T5168, Sigma).

For detection of primary antibodies, secondary antibodies were used: HRP-conjugated anti-rabbit and HRP-conjugated anti-mouse (dilution, 1:2,000) (Cell Signaling Technology). Between each incubation with antibodies, membranes were washed 5 times with TTBS and before applying ECL they were washed 2 times with TBS. Specific bands were visualized by X-ray film and by ImageQuant LAS 4000 (GE Healthcare Biosciences, Pittsburg, PA, USA), after incubation with an enhanced chemiluminescent (ECL) substrate (Western Lightning-ECL, PerkinElmer, Waltham, MA, USA).

Prior to using the anti-hEb peptide antibody for the cell lysates, a series of dilutions and blotting conditions were tested on lysates of U2OS stable monoclonal cell lines expressing RFP-hEb fusion protein as previously described, to optimize the conditions. Antibody was diluted 1:1,000–1:50,000, nitrocellulose vs. PVDF membranes were compared and blocking solutions containing 5% milk, 2% bovine serum albumin and/or 2% horse serum were also compared.

## Results

**Figure 1:**
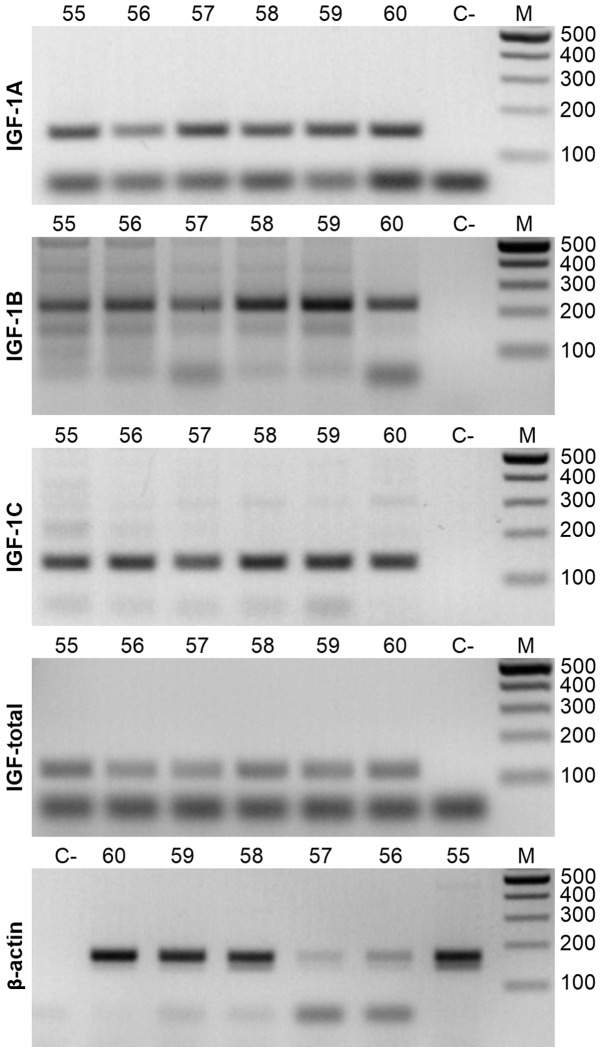
PCR products specific to cDNA of *Igf-1* isoforms, common *Igf-1* sequences and *β-actin*. PCR amplicons were obtained using oligonucleotides described in Table I and resolved in 1.5% agarose gel stained with ethidium bromide. All three *Igf-1* isoforms were amplified with specific oligonucleotides (junction of exons 4, 5 and 6) and oligonucleotides targeting sequences common to all IGF isoforms (junction of exons 3 and 4), *β-actin* was included as a reference gene. A gradient PCR was performed to find optimal annealing temperature; annealing temperatures are shown above each lane. C, negative control; M, 100 bp DNA ladder (New England, Biolabs Inc.). IGF, insulin-like growth factor.

In order to compare *Igf-1* isoform content at the transcript level in cancer cells, qRT-PCR was performed. Total RNA was extracted from HeLa, U2OS, HepG2 and K562 cells and reverse transcribed. Resultant cDNA was analyzed for total *Igf-1* and all isoforms. The specificity of all oligonucleotides used was verified with cDNA derived from mRNA of K562 cells and PCR products were resolved in 1.5% agarose gels. This cell line was used as it is known to have strong levels of *Igf-1* expression (www.proteinatlas.org). Sets of *Igf-1* oligonucleotide pairs previously described in the literature turned out to be below expected specificity within the range of annealing temperatures tested (55–60°C) (data not shown) and for this reason new oligonucleotides for *Igf-1b* and total *Igf-1* were designed to meet expected parameters. Annealing temperature for all subsequent qRT-PCR reactions was set at 60°C (Fig. 1, all panels).

**Figure 2:**
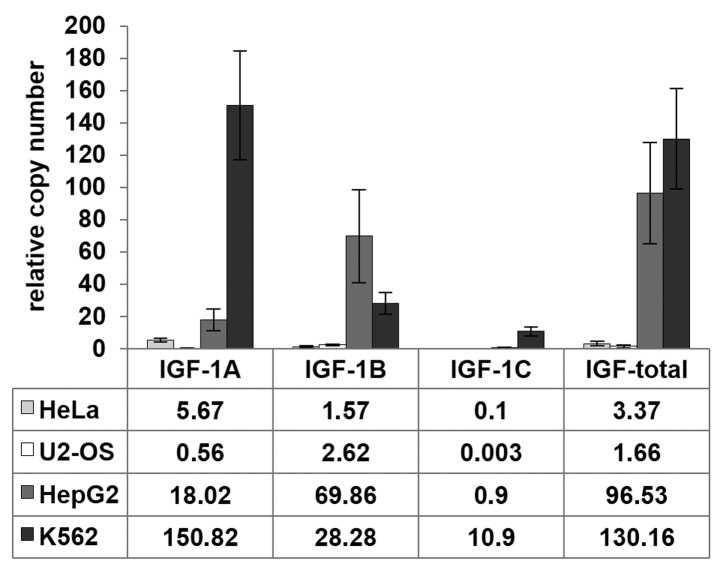
Level of transcripts of different *Igf-1* isoforms. ΔCt was calculated for each *Igf* isoform (*Igf-1a*, *Igf-1b*, *Igf-1c* and total *Igf-1*) as a difference between Ct value for *Igf-1* (*a* or *b* or *c* or total) and Ct value for *β-actin* reference gene. This calculation was performed for each of 3–6 qRT-PCR replicates performed in the study. All Ct values >32 were discarded. Relative copy number: E^ΔCt^, efficiency value (E) was calculated from standard curve traced on the basis of 7 log dilutions of cDNA template (for values see Table I). The outcome was reversed (1/E^ΔCt^) since greater ΔCt means smaller transcript copy number and helps avoid negative numbers and multiplied by a factor of 10^3^ for convenience of greater units. Mean relative copy numbers and standard deviations (bars on the graph) were calculated. IGF-total values were obtained running a separate set of qRT-PCR reactions with specific oligonucleotides recognizing all three isoforms. IGF, insulin-like growth factor.

The qRT-PCR presented notable findings. First, the highest overall relative *Igf-1* expression level was observed in K562 cells when compared to *β-actin* (~130±31.2), slightly lower in HepG2 (~96±31.2) and the lowest in HeLa and U2OS cells (3.4±1.3 and 1.7±1.1, respectively) (Fig. 2). The high expression of *Igf-1* in K562 cells was consistent with database measurements (http://www.proteinatlas.org). *Igf-1a* is the predominant isoform expressed in most tissues (20,21). Similar to the total *Igf-1* results, K562 cells exhibited the highest expression for the *Igf-1a* isoform, where it was 10–300-fold higher than in the 3 other cell lines. *Igf-1b* expression was most pronounced in HepG2 cells, followed by K562 cells. *Igf-1c* levels were the highest in K562 cells, with minimal expression in the three other cells lines with respect to the *β-actin* housekeeping gene. Collectively, we estimated that the predominant isoform in K562 cells was *Igf-1a*, whereas *Igf-1b* appeared to be predominant in HepG2 and U2OS cells. Second, a switch between *Igf-1a* and *Igf-1b* transcript copy number was observed in HepG2 and U2OS cells. In both cell lines, this difference was several fold; for example, in HepG2 cells, a relative copy number of *Igf-1b* transcripts was 69.9±28.6 and of *Igf-1a* transcripts 28.3±6.7 (Fig. 2).

**Figure 3:**
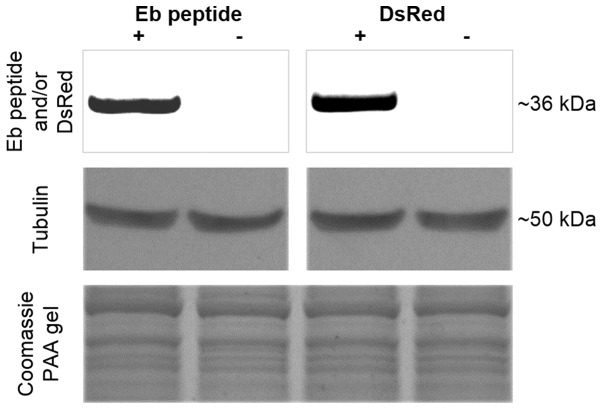
Immunoblotting of hybrid dsRed-hEb protein. Sixty micrograms of protein were loaded in each lane. A 36 kD band can be detected in the lane where U2OS-dsRed-hEb extracts of stable cells is resolved and probed with anti-Eb antibody (panel 1 on the left). The same extract on a different PVDF membrane was probed with anti-dsRed antibody and a 36 kD band is visible (panel 1 on the right). No bands are evident in lanes containing proteins from naïve U2OS extracts. As a loading controls are shown, tubulin (~50 kD) and Coomassie blue staining (panel 2 and 3).

To assess IGF-1B protein expression level and compare it with *Igf-1b* transcript level, a purified rabbit polyclonal anti-Eb peptide antibody was generated. It was first verified using U2OS stable cell lines expressing human Eb-peptide fused with dsRed protein (dsRed-hEb) or naïve U2OS cells. These stable cell lines were described previously (14). They express the fusion protein at a high level as its mRNA is transcribed under control of a strong promoter (P_CMV_). For validation, two separate immunoblots were performed; one with a rabbit anti-Eb antibody and a second with a rabbit anti-dsRed to confirm the presence of the same hybrid protein using a different epitope and the same stable cell line preparation. The results are presented in Fig. 3. It can be concluded that both antibodies revealed a presence of a band of the same molecular mass (36 kD) and anti-Eb peptide antibody specifically recognizes hEb peptide. Thus, the new antibody was specific for hEb.

**Figure 4:**
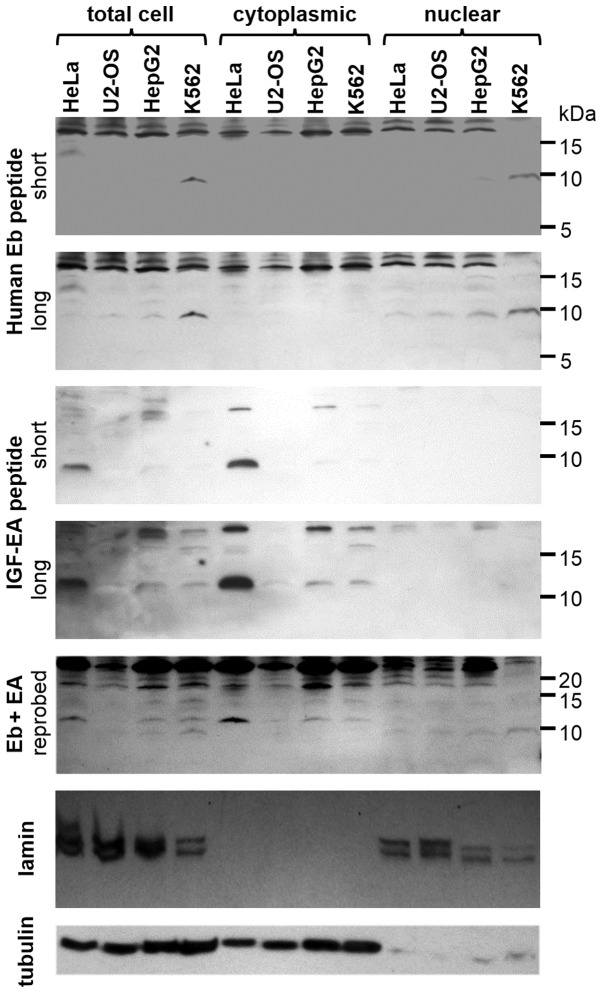
Immunoblotting showing endogenous IGF isoforms in human cancer cells. Sixty micrograms of protein were resolved in each lane. A 9 kD band is present in total cell extract and nuclear fraction from K562 cells and, to a smaller extent, in HepG2 when probed with anti-Eb peptide antibody on the first membrane (short exposure to X-ray film). After longer exposure, this band was also slightly visible in the other two cell lines used in this study (HeLa, U2OS), second panel. It never appeared in cytoplasmic fraction of these cells. A 11–12 kD band was detected when the same membrane was probed with anti-EA antibody, corresponding to human pro-IGF-1A (panel 3). However, this band was evident only in total cell extract and cytoplasmic fraction and never present in the nuclear one (panel 4). Also, another blotting is shown (panel 5), the same membrane already probed with anti-Eb antibody was stripped and reprobed with anti EA-peptide antibody. A merge of both anti-Eb and anti-EA pattern can be observed and a molecular mass difference of 2–3 kD between human Eb-peptide (9 kD) and pro-IGF-1A (11–12 kD). For confirmation of cellular fractionation, 2 different antigens are shown: lamin (nuclear marker), tubulin (cytoplasmic marker) (panels 6 and 7). IGF, insulin-like growth factor.

Since our previous studies were all performed using hybrid dsRed-hEb fusion, we extended our analysis to measure endogenous human Eb peptide and human Ea peptide levels and cellular localization without the aid of epitope tags or fused sequences (14,22). Based on the expression results, we anticipated that the HeLa, U2OS, HepG2, K562 cancer cells would also display a range of IGF-1 protein levels. Immunoblotting with anti-Eb antibody revealed a higher protein content of Eb peptide in K562 cell lysates compared with other cell lines. Only with long exposure times could hEb be detected in the HepG2, U2OS, or HeLa cell lysates. The ~9 kD band size was consistent with hEb cleaved from mature IGF-I. However, in the HeLa cells, a ~16 kD band was also evident, which was the predicted size for pro-IGF-IB. No signal corresponding to human Eb peptide or pro-IGF-IB was observed in the cytoplasmic fraction from any cell line (Fig. 4, panels 1 and 2). However, all cell lines had hEb present in the nuclear fractions, where it was most prominent in the K562 cells, with no evidence of pro-IGF-IB in any nuclear fraction. The 20 kD bands detected by anti-hEb peptide antibody were presumed to be non-specific as there was no Eb-related protein predicted to be that size and the bands were present in all lanes regardless of the cellular fraction.

In parallel, the same preparations (total cell extracts, cytoplasmic and nuclear fractions) were probed with rabbit anti-Ea antibody. A distinct 11–12 kD band corresponding to pro-IGF1A was observed in most samples, in addition to multiple higher molecular weight bands most likely representing glycosylated pro-IGF-IA. However, no hEa peptide (predicted to be ~4 kD) was detected. In contrast to the expression results, pro-IGF-1A levels were the greatest in HeLa cell extracts, with moderate levels of this protein in HepG2 and K562 lysates and virtually no detectable pro-IGF-IA in U2OS cell extracts. All pro-IGF-1A was present in the cytoplasmic fraction and no detection was observed in the nuclear fraction (Fig. 4, panels 1 and 2 vs. panels 3 and 4). In order to demonstrate the presence of both IGF forms, the immunoblots were reprobed with anti-Eb and anti-Ea antibodies simultaneously to ensure that the bands were distinct from each other with respect to size. Although the signals are slightly weaker when compared to immunoblots presented in other panels, both bands corresponding to pro-IGF-1A and hEb peptide can be observed at the expected 2–3 kD difference in size (Fig. 4, panel 5). To demonstrate efficient fractionation of cells, two markers were used for immunoblotting: lamin for nuclear fraction and tubulin for cytoplasmic fraction (Fig. 4, panels 6 and 7). These results confirmed that the cytoplasmic and nuclear fractions were separated.

Upon further analysis, the size of hEb was consistent with the protein being exclusively the E peptide, whereas the size of hEa was only detected as a pro-IGF-I form (attached to mature IGF-I). Thus, the IGF-IA and IGF-IB isoforms not only display different localization and processing in the range of cell lines studied, but there are also clear differences in the relationship between transcription and protein accumulation for the isoforms. Comparisons of IGF-IA and IGF-IB in each cell line showed discordance between RNA and protein levels. In comparison with the expression levels measured by qRT-PCR, it appeared that the K562 cell line exhibited greater post-transcriptional stabilization of hEb than HepG2 cells, for the ~2-fold greater expression of *Igf-1b* in HepG2 vs. K562 cells was reversed in the immunoblotting results, where, at most, hEb levels in HepG2 cells were half of those in K562. For *Igf-1a*, HeLa cells had the greatest intensity of pro-IGF-IA although expression of this isoform was >3–20-fold higher in HepG2 and K562 cells, respectively. Thus, the level of IGF-I production and retention cannot be predicted from measurements of transcription.

Additional blots for total IGF-I and IGF-IC may have helped to clarify the levels of all isoforms in the cell lines. Initial experiments to detect total IGF-I and IGF-IC were unsuccessful, presumably since the endogenous levels of these proteins were below the level of detection by the available antibodies. For instance, several antibodies specific for human IGF-I were tried, but none of them could resolve a clean signal in lanes containing <2 ng human recombinant IGF-I. Thus, we did not pursue the measurements of total IGF-I by immunoblotting. In addition, we did not pursue human Ec as the levels of expression were very low in all cell lines (Fig. 2).

## Discussion

Alternative splicing of the *Igf-1* gene produces several different transcripts, including *Igf-1a*, *Igf-1b* and *Igf-1c* (23). However, understanding the biological significance of these splicing events has been the subject of wide debate for more than 2 decades. It is clear from our study that for *Igf-1a*, *-1b* and *-1c* splice variants, the transcript content does not necessarily reflect the protein content. It is particularly true for pro-IGF-1A expression in HeLa and K562 cells (lower transcript level vs. higher protein content for HeLa cells and vice versa for K562 cells). Several explanations are plausible. First, depending on the cellular context, the post-transcriptional efficiency of one isoform processing into mature IGF-1 protein may vary. Second, in case of IGF-1A protein, processing is even more complex as glycosylated forms can be produced in the cell. We have previously described that both N-sites present in the rodent E peptide extension could carry sugar residues and, depending on cell type, different glycosylation patterns are possible. The proportion of glycosylated pro-IGF1A can be overwhelming as demonstrated with rabbit anti-mouse mature IGF antibody (12, unpublished observations). Closer observation of HepG2 and K562 total cell extract lanes shows bands of molecular mass in the range of 12–15 kD; they could correspond to human glycosylated pro-IGF-1A, which contains one N-glycosylation site. However, due to antigen-antibody affinity reduction by sugar residues, the real protein content of gly-pro-IGF-1A is likely to be largely underestimated. One more explanation to be considered is that pro-IGF-1A protein in HeLa cells is stabilized in direct or indirect interactions with E6 and E7 HPV proteins. It would be consistent with anti-apoptotic properties of these two viral oncogenes that could enhance pro-IGF-1A concentration and activity. These reasons reflect why we do not observe concordant results for IGF-1A between transcript copy number and protein level by western blotting.

It is difficult to compare transcript copy numbers of each IGF isoform, as different cells or tissues were used between studies. Another difficulty is to compare transcript copy numbers of each IGF isoform as different calculations were used between researchers (absolute copy number vs. relative copy number). However, proportions and shifts of IGF splice variants can be compared between studies and within cell lines used in the present study. *Igf-1* expression between normal and cancerous tissues and cells was evaluated by Kasprzak and co-workers and a downshift of the *Igf-1b* content in the favor of *Igf-1a* isoform was reported when colorectal cancer cells and non-tumor tissue were analyzed (24). It is interesting to note that in a previous study, the same group also observed a 10-fold decrease in *Igf-1b* transcript level between cancerous and non-tumor colorectal tissue (25). In our study, a similar *Igf-1* splicing pattern was observed in HeLa and K562 cells where *Igf-1a* transcripts prevailed; however, *Igf-1b* dominated over other isoforms in HepG2 cells. It is not surprising since the fold increase in *Igf-1b* mRNA levels described elsewhere was approximately 3 times greater than the fold increase in *Igf-1a* mRNA levels for liver cells compared with other tissues (20). Furthermore, in a different study, an increase of *Igf-1b* and decrease of *Igf-1a* expression was found when cervical cancer and control cells were compared (26). Thus, it appears that the alterations in *Igf-1* expression and splicing are cell-specific.

There are multiple possible mechanisms by which there is higher human Eb peptide content in K562 compared to HepG2 cells. As there was little pro-IGF-IB found in any cell lines, the pro-peptide could have been cleaved during processing, leading to secretion of mature IGF-I and retention of Eb peptide that becomes sequestered in the nucleus. This would require K562 cells to hold on to significantly more Eb peptide per mole produced, compared to the HepG2 cells. Since the HepG2 cells are derived from liver, which is the primary endocrine organ for IGF-I secretion, it is entirely possible that these cells secrete IGF-I, pro-IGF-I and the E peptides more efficiently that K562. We did not determine how much IGF-I was secreted by any of the cell lines and so this has not been tested directly.

An alternative mechanism for heightened Eb present in the K562 cells, is more efficient Eb peptide uptake from the media, which then accumulates in the nucleus. We know from our previous study that Ea and Ec peptides produced by transfection can be secreted and internalized by neighboring cells (22). One possibility is that pro-IGF-1B is cleaved quickly after translation and there is little pro-IGF-1B present within the cell. Pre-IGF-1 (mature IGF-1 with a signal peptide) would be directed for secretion and Eb-petide containing a nuclear localization signal (NoLS) would be directed to nuclei. Or, human Eb peptide produced in cells from same culture would be secreted as pre-pro-IGF-1 form, cleaved in extracellular milieu and trafficked back to nuclei of target cells. Results presented herein show that K562 and HepG2 cells may be a suitable tool for more extensive studies of IGF-IA and IGF-1B in terms of protein processing and nuclear interactions at endogenous levels. It would be interesting to find out why the IGF-1B isoform and, by extension, human Eb peptide are modulated in different types of cancer cells.

IGF-IC is also known as mechano growth factor (MGF), which has been the focus of much study in skeletal muscle for its potential growth-promoting properties (15,27–29). However, *Igf-1c* levels have also been evaluated in human prostate cancer (PCa) tissues and in human PC-3 and LNCaP cells. IGF-IC/MGF expression was markedly higher in PCa and prostatic intraepithelial neoplasia (PIN) than in normal prostate tissues, while the normal prostate epithelial cells (HPrEC) did not express MGF (30). In our study, IGF-1C transcript copy number was very low in HeLa, U2OS and HepG2 cells, except for K562 cells, in which the proportion of *Igf-1c* transcript vs. total *Igf-1* transcripts was also bigger than in other cells. We did not include the PC-3 cells in the present study, but it is worth noting that PCa growth is regulated by *IGF-IEc* transcript via Ec peptide-specific and IGF-IR/IR-independent signaling (31). However, it is not known if the levels of *Igf-1c* are predominant in human prostate cancer cells and tissues, or if other isoforms are also high.

The differences between the transcript and resultant protein level among different cell types may arise from the efficiency of transcript-protein translation and a number of studies showed that depending on the cellular context, this efficiency may vary (32–34). A good example of how discrepant mRNA and protein levels can be is NOXA expression. NOXA belongs to the Bcl-2 family, promotes activation of caspases and apoptosis. In mantle cell lymphoma (MCL) cells NOXA mRNA was found to be highly expressed, whereas NOXA protein levels were low (35). It has been previously reported that RNA splicing factors were regulated by HPV16 during cervical tumour progression (36); in this context, HPV18 proteins could influence IGF-I splicing pattern in HeLa cells observed in our study. Since viruses use cellular RNA binding proteins for viral RNA processing, it is possible that the splicing of cellular pre-mRNAs is affected by viral infection. For example, the expression of promyelocytic leukemia (PML) isoforms differs in various cell lines or tissues, where PML-I is the dominant form expressed in the brain but not in the liver (37). However, the regulation mechanisms of the cell type- or tissue-specific alternative splicing of PML pre-mRNA have not yet been clarified. In one study, it was demonstrated that the viral protein ICP27 regulates the switching of the PML isoform from PML-II to PML-V by promoting the retention of PML intron 7a (38). Further studies are required to elucidate cells expressing viral proteins, which can interfere not only in splicing events as described earlier, but can also stabilize cellular protein products involved in many important processes: hepatitis B virus × protein (HBx) was reported to stabilize amplified in breast cancer 1 protein (AIB1) and cooperate with it to promote human hepatocellular carcinoma cell invasiveness (39) and it was also reported that cellular proteins were actively stabilized by HSV-1 viral gene products (40). In HeLa cells, such stabilizing activity towards pro-IGF-1A protein could be linked to HPV18 oncoproteins. This hypothesis merits further investigation.

To conclude, it should be emphasized that the presence of human Eb peptide was clearly demonstrated for the first time in nuclear fractions of cancer cells in the present study. Similarly, a novel and sensitive antibody to human Eb peptide proved to be sensitive and specific. Moreover, mouse Ea peptide antibody proved to be suitable for detection of its human counterpart despite four amino acids difference between the two species. A larger picture emerges from our study as well as those of others cited in this manuscript; in different types of cancer, a different IGF isoform and a resultant IGF E-peptide may be down- or upregulated and further involved in growth, proliferation, cell transformation, cancer progression. Viral proteins are also involved in these processes, modifying gene splicing and stabilizing cellular proteins of important actions.

## Figures and Tables

**Table I:** Sequences of oligonucleotides used in real-time PCR for amplification of each of the IGF isoforms using cDNA from human cancer cell lines.

IGF isoform	Sequences (5′-3′)	Product size (bp) (ref)	Slope	qPCR efficiency (E-value %)	R^2^
IGF total	GAGCTGGTGGATGCTCTTCA CATCCACGATGCCTGTCTGA	112 (present study)	−3.4167	96.19	0.9969
IGF-1A	TCGTGGATGAGTGCTGCTTCCG TCAAATGTACTCCCTTCTGGGTCTTG	144 (26)	−3.5875	90.00	0.9987
IGF-1B	GGAGGGGACAGAAGCAAGTC ATCATCTAGCTCCAGCAGGC	224 (present study)	−3.5512	91.25	0.9986
IGF-1C	ACCAACAAGAACACGAAGTC CATGTCACTCTTCACTCCTC	126 (26)	−3.5538	91.15	0.9873
β-actin	TCTGGCACCACACCTTCTAC GATAGCACAGCCTGGATAGC	169 (26)	−3.5827	90.20	0.9896

Relative copy number was calculated with regard to *β-actin* amplification. IGF, insulin-like growth factor.
